# Assessment of Tomato (*Solanum lycopersicum* L.) Producers’ Exposure Level to Pesticides, in Kouka and Toussiana (Burkina Faso)

**DOI:** 10.3390/ijerph15020204

**Published:** 2018-01-25

**Authors:** Diakalia Son, Fabrice K. B. Zerbo, Schémaeza Bonzi, Anne Legreve, Irénée Somda, Bruno Schiffers

**Affiliations:** 1 Agrosystèmes et Ingénierie de l’Environnement (Sy.N.A.I.E), Unité Santé des Plantes du Laboratoire Systèmes Naturels, Institut du Développement Rural (IDR), Université Nazi Boni (UNB), 01 BP 1091 Bobo-Dioulasso, Burkina Faso; benzerbo@yahoo.fr (F.K.B.Z.); ouakobonzi@yahoo.fr (S.B.); ireneesomda@yahoo.fr (I.S.); 2 Gembloux Agro-Bio Tech/ULIEGE—Pesticide Science Laboratory, Passage des Déportés 2, 5030 Gembloux, Belgium; bruno.schiffers@ulg.ac.be; 3 Phytopathology, Earth and Life Institute, Catholic University of Louvain, Croix du Sud, 2 bte L7.05.03, B-1348-Louvain-la-Neuve, Belgium; anne.legreve@uclouvain.be

**Keywords:** tomatoes, pesticides, risk assessment, UK-POEM, producers, Burkina Faso

## Abstract

To assess producers’ exposure level to pesticides in vegetable production in Burkina Faso, a study was carried out in 2016 and 2017 among 30 tomato producers in the municipalities of Kouka and Toussiana. Eighteen (18) commercial formulations were identified, with more than 50% of pesticides destined for cotton production. Eleven active substances have been identified and the most frequently used are λ-cyhalothrin (35%), acetamiprid (22%) and profenofos (13%). The most commonly used chemical families are pyrethroids (28%) and organophosphates (18%). The study revealed a low level of training for producers, a high use of pesticides according to the Frequency Treatment Indicator, and a very low level of protection used by producers. The Health Risk Index shows that active substances such as methomyl, λ-cyhalothrin and profenofos present very high risk to operators’ health. Based on the UK-POEM model, the predictive exposure levels obtained varied from 0.0105 mg/kg body weight/day to 1.7855 mg/kg body weight/day, which is several times higher than the Acceptable Operator Exposure Level. However, the study also shows that exposure can be greatly reduced if the required Personal Protective Equipment is worn. Producers’ awareness and training on integrated pest management are necessary to reduce the risks linked to the pesticides use in Burkina Faso.

## 1. Introduction

In Burkina Faso, tomato cultivation only takes second place to onions with a production of 289,572 tons on an area of 11,766.4 ha during the 2013–2014 vegetable season [1]. However, this production is subject to many constraints, including pest pressure (*Bemisia tabaci* Gennadius, *Helicoverpa armigera* Hübner, *Tuta absoluta* Meyrick), which forces producers to intensify chemical treatments beyond suggested recommendations by overdosing or increasing the number of treatments [2,3,4]. Although plant protection products (PPP) enable satisfactory results in agricultural production, their use is risky to human health, the environment and non-target organisms [5,6,7]. This risk is higher because of the use in vegetable production of toxic and highly concentrated PPPs intended to combat pests in cotton without appropriate protective equipment [8,9]. Surveys on phytosanitary practices in tomato production in Burkina Faso show that more than 70% of producers do not take adequate protective measures during PPP use [9]. Several studies have shown that skin exposure remains the main risk allowing pesticide penetration into the human body [7,10]. This could explain the adverse effects experienced by producers (skin irritations, hot flushes, headaches, etc.) following pesticide application [11,12,13]. 72% of 316 producers, surveyed on their phytosanitary practices in tomato production in Burkina Faso, complained of acute poisoning during or after pesticide application [9]. In addition to these acute effects, the chronic effects of pesticides on producers’ health, such as cancer, neurological diseases and reproductive disorders, have been highlighted by several authors [7,14]. On top of all this, a misuse of PPPs can also lead to consumer exposure (residues in food and water), environmental contamination (water and soil), emergence of resistant strains and auxiliary fauna destruction [15,16].

Compared to cotton production, few studies have evaluated the potential exposure of Burkina Faso vegetables producers’ to pesticides [7,11]. However, the significant development of vegetables gardening in Burkina Faso and the intensification of phytosanitary treatments, especially in tomato production, which have not been preceded by any impact study beforehand, justify this study considering that measuring the risk to operators is an obligation when registering PPPs [17,18].

It is in this context that this field study marked by close monitoring of some tomato producers surveyed in 2015–2016 [9], was carried out in 2016 and 2017, not only to further characterize their practices, assess the frequency and intensity of phytosanitary treatments, but also to assess the potential exposure of producers to these pesticides.

The importance of this paper after that on pytosanitary practices of tomato producers in Burkina Faso [9], is intended to once more draw the attention of producers and authorities to the risks associated in pesticides use and the necessity to apply the better phytosanitary practices.

## 2. Materials and Methods

### 2.1. Observation Sites

**Figure 1:**
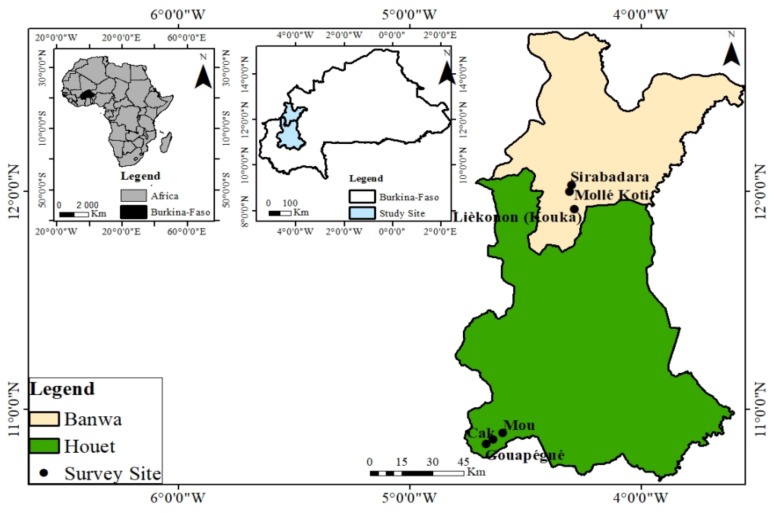
Location of the six observation sites of phytosanitary practices of tomato producers in the communes of Kouka and Toussiana (Burkina Faso).

In order to characterize producers’ phytosanitary practices, surveys and close monitoring were made in the provinces of Banwa in 2016 and Houet in 2017 (Western Burkina Faso). In each province, 15 tomato producers were randomly selected from the six main production sites located in two communes (Kouka and Toussiana) (Figure 1). 

These volunteer producers were selected from those surveyed in 2015–2016 on their phytosanitary practices [9] to follow end-to-end their phytosanitary practices (pesticides used, dosage, Personal Protective Equipment (PPE) worn during treatments, etc.) since the transplanting of their tomato plants until the harvest, by systematically recorded all activities and behaviors during pesticides use.

### 2.2. Observations and Measurements

In order to assess producer’s dependency and exposure to pesticides, observations and measurements were made on 30 producers during phytosanitary applications. Those observations were based on:

- The pesticides used by the producer (commercial name of the PPP, active substances, recommended dose and actual dose used);
- The personal protective equipment (PPE) worn by the producer;
- The quality of the material (measuring container used for dosing and spraying);
- The negligent behaviour (eating, smoking, urinating, ...) during the application;
- The sanitation measures taken after pesticide application immediately washing hands and feet.

The measures related to the PPP application conditions are:

- The treated area during each application, using a Global Positioning System (GPS);
- The doses and volumes of spray mix used, with graduated receptacles;
- Preparation, application and rinsing times of the equipment, using a chronometer;
- Temperature (°C) and wind speed (m/s), using a CFM/CMM Thermo-Anemometer, model DT-619 (Ruby Electronics, Saratoga, CA, USA);
- Air humidity, with a digital thermo-hygrometer using a Profi-Thermo-Hygrometer, TFA (Dostmann GmbH + Co. KG, Zum Ottersberg, Germany).

### 2.3. Computation of the Health Risk Indexes

Based on the information collected on the phytosanitary practices of producers and data indexed in databases on the pesticides’ toxicological properties (SAgE pesticides), the health risk indexe (HRI) was calculated using the Quebec-IRPeQ pesticides risk indicator developed by the Quebec National Institute of Public Health (INSPQ), the Quebec Ministry for Sustainable Development, the Environment and the Parks (MDDEP) and the Quebec Ministry for Agriculture, Fisheries and Food (MAPAQ) [19]. The toxicological data used to compute the indexes have been collected in various databases [European Union Pesticide Database, SAgE Pesticides (Canada), Agritox and INERIS (France), etc.]. These toxicological properties have been classified according to their Classification, Labeling and Packaging (CLP) by the EU-Pesticides Database [20]. The choice of the indicator of risks of the pesticides (IRPeQ) to calculate HRI was made by considering the availability of the tool, its ease of use depending on data accessibility for the most active ingredients. It was used by [21] and by [22] in Benin and Tunisia, enabled the evaluation and toxicity comparison of various active substances. HRI calculation integrates acute toxicity values (oral LD_50_, dermal LD_50_, inhalation LC_50_, eyes and skin irritation, sensitization), chronic toxicity values (carcinogenic risks, reproductive and developmental risks, genotoxicity and potential endocrine disruption) modulated by a factor related with persistence and bio-accumulation of active substances in the human body (biocentration factor or BCF). It also takes into account the concentration, the formulation type and the application dose. HRI help to rank the toxicity of pesticide in order to choose those that are less harmful to human health [19]. It is calculated as follows:

$$HRI=\frac{TRI\times FPf\times FCP}{10}$$

$$HRI_{PPP}=\sum HRI_{Active\;substance}$$

with:

- *HRI_active substance_* = Health risk index for the active substance;
- *TRI* = Toxicological risk index of the active substance = [Σ of acute toxicity points + (Σ of chronic toxicity points × *FPer*)]^2^. To obtain a greater distribution of values and to highlight more the pesticides presenting at higher risk, the sum of the variables was squared;
- *FPer* = Factor taking into account the environmental persistence, (based on TD_50_ in soil) or the bioaccumulation potential in humans (*BCF* value). It varies from 1 to 2.5;
- *FPf* = Weighting factor related to formulation type. It varies from 1 to 2 depending on the potential contamination via the formulation (respectively low risk and high risk);
- *FCP* = Compensation factor to account for the active substance concentration in the end-use product and the applied dose (concentration × recommended dose/ha);
- 10 = Quotient to obtain an *HRI* of an acceptable order of magnitude, as the value obtained may be very high for some active substances with high *TRI*.

The criteria for acute and chronic toxicity of the active substances are weighted by points [19].

### 2.4. Treatments Frequency and Intensity Indicator

The treatment frequency index (*TFI*) corresponds to the ratio between the applied dose and the dose recommended on the label, taking into account the area of the treated plot [23,24]. Each application is regarded as a treatment, even in the case where the product is used in divided doses. A mixture of two products applied during the same passage also counts for two treatments:

$$TFI_{Treatment}=\frac{DU\times St}{RD\times ST}$$

with:

- *TFI_Treatment_* = *TFI* calculated during each PPP application;
- *DU* = Dose used by the producer during each loading of the sprayer;
- *RD* = Recommended dose of the PPP;
- *St* = Area of the plot treated during each application;
- *ST* = Total field area.

The *TFI* per plot corresponds to the sum of *TFIs* per treatment throughout the production cycle:

$$TFI_{Plot}=\sum TFI_{treatment}$$

The calculated *TFI* is compared to the regional or national reference *TFI*. In case of absence of a reference *TFI*, it is compared with the 70th percentile of the *TFI* for the crop considered after surveying a minimum of 30 plots [25].

### 2.5. The Model Used to Assess Producers Dermal Exposure

**Figure 2:**
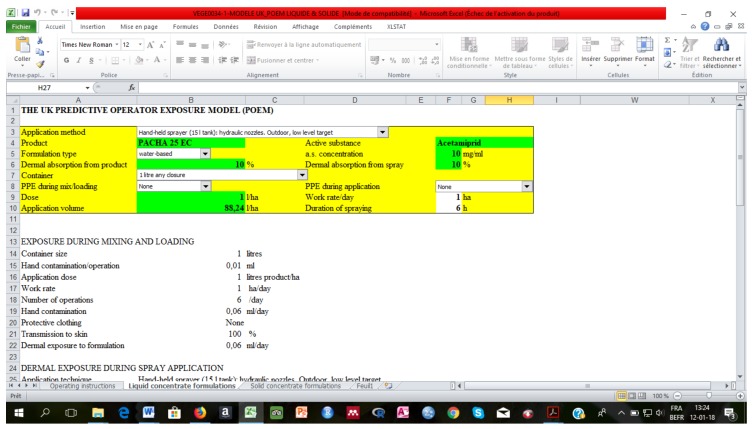
Screenshot of the UK-POEM model spreadsheet.

In order to estimate the potential exposure level (PE, in mg/kg body weight/day), the British model or the UK Predictive Operator Exposure Model (UK-POEM) was used. It is presented on an Excel sheet (Figure 2). Parameters such as the application method, the formulation and the PPP’ concentration, the personal protective equipment (PPE), the dose and the volume of application were used in the model in accordance with local practice. This model was also used by other authors [7,26,27]. All the required parameters in this model are presented in the Table 1.

The total predictive exposure is the sum of dermal and inhalation exposure during mix/loading (mainly through hands contact) and the spraying (droplets received all over the body). The predictive exposure was estimated for two scenarios: without PPE, to the most common scenario in Burkina Faso, and with PPE (mask, gloves and coverall). The calculation is made by active substance and for each pesticide.

### 2.6. Risk Characterization

The risk for each active substance used by the producers was characterized by comparing the predictive exposure value expressed in mg/kg body weight/day with Acceptable Operator Exposure Level (AOEL). When this value is lower than the value of AOEL, the risk may be considered acceptable. If the risk is considered unacceptable for the market gardener, mitigation measures should be recommended.

### 2.7. Statistical Analysis 

The treatment frequency index (*TFI*) values of the different sites, after a logarithmic transformation, were analyzed by a single factor ANOVA after verification of the normality and the homoscedasticity of the data (R 3.3.3 software (Manufacturer by Kurt Hornik., Welthandelsplatz, Austria) [29]). Moreover, an HSD Tukey test of structuring of averages was carried out.

## 3. Results

### 3.1. PPP Used by the Surveyed Producers and Toxicity of Active Substances

PPP and active substances used by producers are listed in Table 2. Eighteen (18) commercial formulations, consisting of 73% insecticides, 18% fungicides and 9% insecticides-acaricides, were identified. Five of these formulations are not approved by the Sahelian Pesticides Committee (SPC), which is the only office of the Permanent Inter-State Committee for Drought Control in the Sahel (CILSS) that regulates the use of pesticides in its Member States. The usage rate of pesticides registered for cotton but used on tomatoes was 54% in Kouka and 25% in Toussiana. Eleven active substances were identified; the most frequently used are λ-cyhalothrin (35%), acetamiprid (22%), profenofos (13%) and cypermethrin (12%). The most widely used chemical families are pyrethroids (28%), organophosphates (18%) and carbamates (18%).

The calculation of the health risk index revealsed that methomyl, λ-cyhalothrin, profenofos and chlorothalonil are active substances that showed the highest risks of poisoning (Table 3). Both profenofos and indoxacarb present the highest risk of acute and chronic toxicity.

The PPPs that pose the greatest risk to human health are: POLYTRINE 336 EC, TROPISTAR P 186 EC, AVAUNT 150 SC and LAMBDACAL P 636 EC (Table 4). These PPPs are normally recommended for cotton production.

### 3.2. Level of Education and PPE Worn by Tomato Producers

Among the producers surveyed, 70% received no education and only 13% received training in plant protection. The lowest level of education was observed in the Township of Kouka (80% of surveyed producers). There were no PPE available in compliance with phytosanitary applications (mask, gloves, protective clothing) used by the surveyed producers (Table 5). The few producers who used masks and gloves, usually made of cloth, wore them during pesticide application only and not also during the preparation of the spray mix, despite the risk of inhaling concentrated pesticide vapors.

### 3.3. Status of Sprayers and PPP Dosage

**Figure 3:**
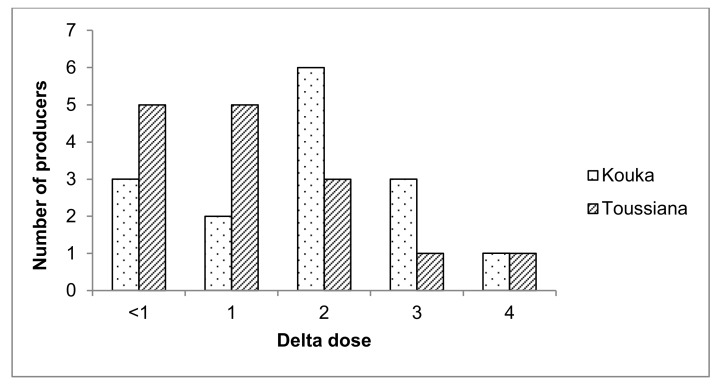
Pesticide dosage profile by the 30 tomato producers surveyed in Kouka and Toussiana (Burkina Faso). Delta dose is the ratio of the dose used by the producer (L or g) to the recommended dose for the treated area.

30% of the backpack sprayers used by the producers surveyed are in poor condition and leak during pesticide application. Few producers (two from Kouka and five from Toussiana) meet the recommended dose of PPP. 27% (three producers from Kouka and five from Toussiana) were below the recommended dose and 50% of surveyed producers overdose their PPP (Figure 3).

### 3.4. Intensity of Treatment and Observed Carelessness 

**Figure 4:**
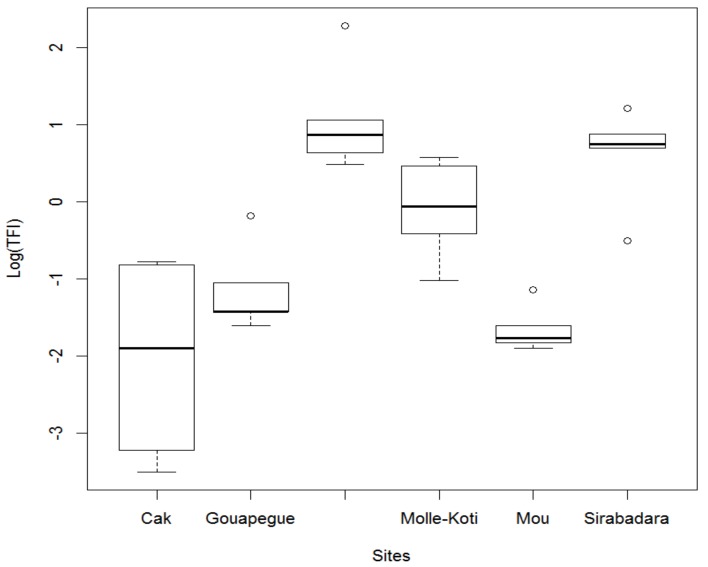
Comparison of TFI of the six sites observed in the communes of Kouka and Toussiana (Burkina Faso).

The *TFI* indicates a high use of pesticides in the commune of Kouka, with nine producers above the 70th percentile compared to four producers in Toussiana (Table 6). Figure 4 shows a highly significant difference between sites (*p* ≤ 0.001). However within the same township, no significant difference was observed between the sites. The average number of treatments per tomatoes production cycle is 11.93 ± 2.58 in Kouka and 5.33 ± 1.68 in Toussiana.

**Figure 5:**
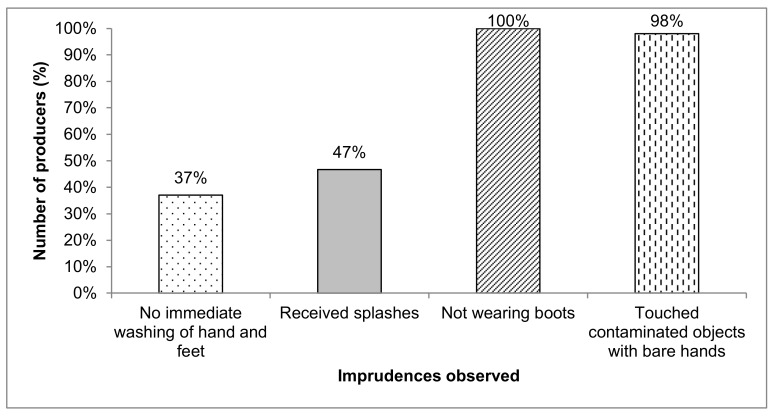
Types of carelessness observed during treatments amongst 30 producers in Kouka and Toussiana (Burkina Faso).

As for the carelessness observed which may favor the rapid exposure of the operator to pesticides, they are represented in Figure 5. All market gardeners surveyed used their bare hands to manipulate the product packaging and contaminated objects (measuring instruments, lance, nozzles) and 43% did not wash their hands before urinating during the application of PPP.

### 3.5. Exposure Risks and Health Effects Witnessed by Producers.

Results in Table 7 show that producers could be highly exposed to pesticides, especially in Kouka Township. Apart from cypermethrin and acetamiprid, all used active substances presented unacceptable risks to the operator with estimated exposure levels exceeding several times the acceptable operator exposure level (AOEL). The potential values for dermal exposure during mixing, loading and spraying when producers work without PPE range from 0.0136 mg/kg bw/day (acetamiprid) to 1.7855 mg/kg bw/day (chlorothalonil) in Kouka and from 0.0105 mg/kg·bw/day (acetamiprid) to 0.2914 mg/kg bw/day (profenofos) in Toussiana. λ-Cyhalothrin is more likely to be exposed at more than 2000% of AOEL in both townships. However, when using complete PPE (wearing of mask, gloves and protective clothing), the risk of exposure is reduced by more than 800 times with λ-cyhalothrin. In terms of the effects felt by producers during or after pesticide use, 57% of producers reported that they felt certain health effects such as skin irritation (23%), eye diseases (19%), nasal discharge and coughing (11%).

## 4. Discussion

The results show a high use of PPP normally recommended for cotton production being used tomatoes, especially in the Kouka township, and that they present a high risk to health according to the calculated HRI. Schiffers and Mar [8] reported that these PPP are not recommended in vegetable production because of their high toxicity and high concentration of active substances. Pyrethrinoids are the most commonly used, and several authors have reported the resistance of the main tomato pests like *B. tabaci*, *H. armigera* and *T. absoluta* to the insecticides of this chemical family [30,31,32]. This choice leads to an intensification of treatments and consequently to an increased risk of poisoning from exposure. Among the formulations used, 75% are liquid. In general, the substances present in these formulations are more easily absorbed through the skin and other tissues than solid formulations [33]. According to Berenstein et al. [34], exposure from liquid PPP is 22 to 62 times higher than that of solid PPP.

While the use of PPP requires a minimum amount of knowledge to work safely, the results of the study showed a low level of education and training of the producers. Unable to read or write, producers’ capability to understand and follow instructions mentioned on the labels (dose to be applied, safety instructions, PPE to be worn, hygiene, etc.) is limited, which increases the risks of exposure. According to Jallow et al. [35], insufficient knowledge, the influence of retailers, and the lack of access to alternative pest management methods are pushing producers to use PPP. On the other hand, the higher the level of education and training, the lower the exposure [35,36]. Therefore, after two years of Integrated Pest Management (IPM) training, [37] found that trained farmers used less pesticide, spent less money on pest management, and endured less exposure to pesticides. In Mali, after 8 years of IPM training, the use of hazardous insecticides by cotton producers decreased by 92.5% compared to those who had not received training [38].

Compliance with the type of PPE depending on the toxicity of the pesticide used, the formulation (liquid, powder or granules) and the type of activity (mixing, loading or spraying), enables the pesticide applicator to reduce exposure. A study conducted in lemon trees revealed that dermal exposure would be reduced by 27% by using gloves, 38% by protective clothing and 65% by gloves and protective clothing [39].

However, our study showed a low level (or absence) of producer protection. The few producers who used masks and gloves wore them during the application of pesticides but not during the preparation of the mixture when the greatest exposure occurs because the product is handled in the concentrated state and the risk of inhalation of concentrated pesticide vapors remains high [8,40,41]. 64% of hand contamination occurred during the mixing-loading phases [36], 20% of producers’ wore short-sleeved clothing and shorts, while several studies have highlighted heavy contamination of legs and forearms during pesticide application [36,42,43,44]. Another factor favoring producers’ exposure to pesticides is the use of defective sprayers that leaked during the application of pesticides. This increases the contamination rate, because even in the normal state (absence of leaks), contamination via the hands is 25% and 50% by the legs with the backpacker if there is no adequate protection [45].

In addition to the lack of wearing of PPE and the use of defective sprayers, the intensification of treatments (increased doses and number of treatments) also favors the exposure of producers to pesticides. According to several authors [46], dermal and respiratory exposure is proportional to the application rate and the frequency of application. According to Baldi et al. [47] spraying is responsible for 50% of the total daily exposure. Failure to comply with hygienic measures such as washing hands before urinating during PPP application is also a very important risk factor for pesticide contamination of producers. In accordance with Poet [48], pesticides are absorbed 12 times faster by the genitals compared to the forearms.

The weather conditions at the time of application, such as temperature and air humidity, can affect the volatility of the product and the rate of sweating of human body [46,49,50]. High temperatures cause excessive sweating to promote rapid penetration of the product into the body and winds above normal (1 and 2 m/s) can transport the product out of the targeted zone and cause contamination of the applicator by the pesticide [46,51,52]. The meteorological conditions recorded at the level of the two localities during the application of pesticides are within the recommended ranges in the exposure of the operators. Pesticide applications are carried out either very early in the morning or in the evening when the weather conditions are favorable.

λ-Cyhalothrin presents an unacceptable risk of toxicity to producers, where it is most used in market gardening production in Burkina Faso and in the subregion [9,12,27,53,54]. This active substance is extremely toxic to humans (irritation of eyes, skin, colds and coughs) [55,56]. This could largely explain the malaise felt by 57% of the producers monitored. In the short term, it is neurotoxic (ataxia, tremors, occasional convulsions), but in the long term it is not carcinogenic or genotoxic and has no effect on reproduction and development but rather causes a decrease in body weight [55]. However, with mask, gloves and protective clothing, the risk of exposure can be reduced by more than 800 times, thus demonstrating the importance for the operator to wear complete PPE to reduce pesticide contamination [7,57,58].

## 5. Conclusions

The results of this study showed an intensification of pesticide use in tomato production in Burkina Faso with high exposure risks. According to the study, there is little training of producers in the use of plant protection products in relation to their inappropriate practices (use of highly toxic pesticides, overdose, no use of PPE, etc.). Apart from acetamiprid and cypermethrin, all the active substances exceeded the exposure values of their acceptable exposure level (AOEL) for the operator. λ-Cyhalothrin, which is the most widely used active ingredient in vegetable production in Burkina Faso, and in the two localities studied, present a particularly high risk of exposure for the producers. However, this exposure can be reduced by 800 times if recommended PPE were used. To promote the rational management of pesticides and limit their impact on human health and the environment in Burkina Faso, it is necessary to reduce and control the use of pesticides by:

- Raising awareness among producers to the risks and the training on the recognition of pests and auxiliaries to be respected;
- Increasing popularity of biopesticides and alternative methods, as well as the promotion of integrated pest management;
- Providing training based on the rules of best practice for the use of pesticides, emphasizing safety instructions and the importance of the use of protective equipment.

## Figures and Tables

**Table 1:** Parameters used in the UK-POEM model to estimate producers’ potential exposure to pesticides.

Parameters Used in the UK-POEM Model	Details
Application method	Backpack sprayer (15 L tank)
Formulation type	EC, SC or WP
Dermal absorption from product	10% default value [28]
Absorption through inhalation	100% default value [28]
Container	1 L, any closure
Personal Protective Equipment (PPE)	Scenario 1: none Scenario 2: mask, gloves and coverall
Surface treated/day	1 ha (default value)
Duration of spraying	6 h (default value)
Operator weight	60 kg (WHO conventional body weight)

**Table 2:** List of PPPs used by 30 producers in tomato production in Kouka and Toussiana (Burkina Faso).

Trade Name of PPP	Area of Use	Formulation	Active Substances	Chemical Families	SPC Approval	WHO Class
ACARIUS 018 EC	Vegetables	EC	Abamectin (18 g/L)	Avermectin	Yes	I
AVAUNT 150 SC	Cotton	CS	Indoxacarb 150 g/L	Carbamates	Yes	-
BIOK 16	Vegetables	WP	Bt var. kurstaki: 2–4% (16,000 UI/mg)	Bacillaceae	Yes	III
COGA 80 WP	Vegetables	WP	Mancozeb (800 g/kg)	Carbamates	Yes	U
CONQUEST 176 EC	Cotton	EC	Cypermethrin (144 g/L) + Acetamiprid (32 g/L)	Pyrethroids + Neonicotinoids	Yes	II
DUEL CP 186 EC	Cotton	EC	Cypermethrin (36 g/L) + Profenofos (150 g/L)	Pyrethroids + Organophosphates	None	II
EMA 19.2 EC	Cotton	EC	Emamectin benzoate (19.2 g/L)	Avermectin	Yes	II
EMACOT 019 EC	Cotton	EC	Emamectin benzoate (19 g/L)	Avermectin	Yes	II
EMIR FORT 104 EC	Cotton	EC	Cypermethrin (72 g/L) + Acetamiprid (32 g/L)	Pyrethroids + Neonicotinoids	Yes	II
JUMPER 75 WG	Vegetables	WG	Chlorothalonil (750 g/kg)	Chloronitrile	Yes	U
K-OPTIMAL	Vegetables	EC	λ-Cyhalothrin (15 g/L) + Acetamiprid (20 g/L)	Pyrethroids + Neonicotinoids	Yes	II
LAMBDA POWER	Vegetables	EC	λ-Cyhalothrin (25 g)	Pyrethroids	None	II
LAMBDA SUPER 2.5 EC	Vegetables	EC	λ-Cyhalothrin (25 g)	Pyrethroids	None	II
LAMBDACAL P636EC	Cotton	EC	Λ-Chyhalothrine (36 g/L) + Profenofos (600 g/L)	Pyrethroids + Organophosphates	Yes	II
PACHA 25 EC	Vegetables	EC	Λ-Cyhalothrin (15 g/L) + Acetamiprid (10 g/L)	Pyrethroids + Neonicotinoids	Yes	II
POLYTRINE 336 EC	Cotton	EC	Cypermethrin (36 g/L) + Profenofos (300 g/L)	Pyrethroids + Organophosphates	None	II
SAVAHALER	Vegetables	WP	Methomyl (250 g/kg)	Carbamates	Yes	Ib
TROPISTAR 336 EC	Cotton	EC	Cypermethrin (36 g/L) + Profenofos (300 g/L)	Pyrethroids + Organophosphates	None	II

EC = Emulsifiable concentrate; WP = Wettable powder; CS = Concentrated suspension; WG = Water-dispersible granules; Class I: extremely/highly hazardous; Class Ib: very hazardous to humans; Class II: moderately hazardous; Class III: slightly hazardous, Class U: Unlikely to present a hazard to humans under normal use conditions.

**Table 3:** Value of the parameters used in the calculation of the Health Risk Index (*HRI*) and the toxicity of the active substances used for tomato protection in Kouka and Toussiana (Burkina Faso).

Active Substances	Use Rate	Σ of Acute Toxicity Points	Σ of Chronic Toxicity Points	*FPer*	*TRI*	*FPf*	*FCP*	Points Allocated to *HRI*	CLP Classification
Profenofos	12%	20	18	1	1444	2	0.73	209.4	H302, H312, H332
Indoxacarb	6%	15	18	1.5	1764	2	0.52	183.2	H301, H317, H332, H372
Methomyl	2%	26	4	1.5	1024	2	0.78	160.0	H300
Mancozeb	1%	10	6	1	256	2	2.00	102.4	H317, H361d
Cypermethrin	13%	18	4	2	676	2	0.53	71.3	H302, H332, H335
Chlorothalonil	1%	20	0	1	400	1	1.63	65.0	H317, H318, H330, H335, H351
Abamectin	4%	19	4	1.5	625	2	0.51	64.2	H300, H330, H361d, H372
λ-Cyhalothrin	35%	25	0	2	625	2	0.50	64.0	H301, H312, H330
Emamectin benzoate	5%	17	0	1	289	2	0.51	29.3	Unclassified
Acetamiprid	26%	9	2	1	121	2	0.52	12.5	H302
*Bacillus thuringiensis*	1%	Unclassified	Unclassified	Unclassified	Unclassified	Unclassified	Unclassified	Unclassified	Unclassified

*FPer* = Factor taking into account the environmental persistence or the bioaccumulation potential in humans; *TRI* = Toxicological risk index of the active substance; *FPf* = Weighting factor related to formulation type; *FCP* = Compensation factor to account for the active substance concentration in the end-use product and the applied dose; *HRI* = Health risk index for the active substance; H300 = Fatal if swallowed; H301 = Toxic if swallowed; H302 = Harmful if swallowed; H312 = Harmful in contact with skin; H317 = May cause an allergic skin reaction; H318 = Causes serious eye damage; H330 = Fatal if inhaled; H332 = Harmful if inhaled; H335 = May cause respiratory irritation; H351 = Suspected of causing cancer; H361d = Suspected of damaging fertility or the unborn child; H372 = Causes damage to organs through prolonged or repeated exposure.

**Table 4:** Decreasing ranking of the toxicity of pesticides used by tomato producers in Kouka and Toussiana (Burkina Faso) according to the Health Risk Index (*HRI*).

Trade Name of PPP	Active Substances	Points Allocated to *HRI*
POLYTRINE 336 EC	Cypermethrin + Profenofos	280.63
TROPISTAR P 186 EC	Cypermethrin + Profenofos	280.63
LAMBDACAL P 636 EC	Lambda-cyhalothrin + Profenofos	273.29
AVAUNT 150 SC	Indoxacarb	183.15
SAVAHALER	Methomyl	160.00
DUEL CP 186 EC	Cypermethrin + Profenofos	136.05
COGA 80 WP	Mancozeb	102.40
CONQUEST 176 EC	Acetamiprid + Cypermethrin	83.71
EMIR FORT	Acetamiprid + Cypermethrin	83.71
K-OPTIMAL	λ-Cyhalothrin + Acetamiprid	76.37
LAMANET 46 EC	λ-Cyhalothrin + Acetamiprid	76.37
PACHA 25 EC	λ-Cyhalothrin + Acetamiprid	76.37
JUMPER 75 WC	Chlorothalonil	65.00
ACARIUS 018 EC	Abamectin	64.19
LAMBDA POWER	λ-Cyhalothrin	63.91
LAMDA SUPER 2.5 EC	λ-Cyhalothrin	63.91
EMA 19.2 EC	Emamectin benzoate	29.32
EMACOT 019 EC	Emamectin benzoate	29.32
BIO K 16	*Bacillus thuringiensis*	Unclassified

**Table 5:** Level of PPE adoption of 30 producers during the use of pesticides in tomato protection in Kouka and Toussiana (Burkina Faso).

PPE/Clothing	SS and S	SS and T	LS and T	Total
No protection	20%	43%	7%	70%
Mask	0%	7%	17%	24%
Mask + Gloves	0%	3%	3%	6%
Total	20%	53%	27%	100%

SS = short sleeves; S = shorts; LS = long sleeves; T = trousers.

**Table 6:** *TFI* values for tomato protection in Kouka and Toussiana (Burkina Faso).

Communes	Number of Producers	*TFI* Minimum	Average *TFI*	TFI at the 70th Percentile	Maximum *TFI*
Kouka	15	0.36	2.29 ± 2.24	2.32	9.78
Toussiana	15	0.03	0.27 ± 0.20	0.30	0.83
Total	30	0.03	1.28 ± 1.87	1.67	9.78

**Table 7:** Decreasing ranking of the exposure of tomato producers in Kouka and Toussiana (Burkina Faso).

Active Substances	LD_50_ (Dermal) (mg/kg·bw/day)	Number of Producers Using This Active Substance		Operator Exposure (mg/kg·bw/day): Unprotected		Operator Exposure (mg/kg·bw/day): Complete Protection		AOEL (mg/kg·bw/day)	% AOEL (Unprotected)		% AOEL (Complete Protection)	
		Kouka	Toussiana	Kouka	Toussiana	Kouka	Toussiana		Kouka	Toussiana	Kouka	Toussiana
Chlorothalonil	>10,000	1	0	1.7855	-	0.3978	-	0.0090	19,839%	-	4420%	-
Methomyl	>2000	0	2	-	0.1738	-	0.0204	0.0025	-	6950%	-	816%
Mancozeb	>5000	1	0	1.6905	-	0.2371	-	0.035	4830%	-	677%	-
Emamectin benzoate	>2000	2	2	0.0129	0.0144	0.0018	0.0018	0.0003	4314%	4800%	611%	583%
Lamda-cyhalothrin	632	14	12	0.0172	0.0151	0.0021	0.0021	0.0006	2732%	2410%	339%	339%
Indoxacarb	>5000	5	0	0.0714	-	0.0080	-	0.0040	1785%	-	199%	-
Profenofos	>2000	9	1	0.2753	0.2914	0.0319	0.0442	Unavailable	-	-	-	-
Abamectine	>2000	0	3	-	0.0151	-	0.0021	0.0025	-	604%	-	83%
Cypermethrin	>4920	9	2	0.0595	0.0407	0.0065	0.0058	0.0600	99%	68%	11%	10%
Acetamiprid	>2000	10	12	0.0136	0.0105	0.0016	0.0015	0.0700	19%	15%	2%	2%

The LD_50_ is the amount of a single-dose administered at one time that causes the death of 50% (half) of a group of test animals.
